# Role of Perception, Health Beliefs, and Health Knowledge in Intentions to Receive Health Checkups among Young Adults in Korea

**DOI:** 10.3390/ijerph192113820

**Published:** 2022-10-24

**Authors:** Mi-Kyoung Cho, Yoon-Hee Cho

**Affiliations:** 1 Department of Nursing Science, Chungbuk National University, Cheongju 28644, Korea; 2 Department of Nursing, College of Nursing, Dankook University, Cheonan 31116, Korea

**Keywords:** mass screening, young adult, health belief model, attitude to health, knowledge

## Abstract

Regular health checkups allow early treatment when problems occur and prevent disease progression, but the rate of health checkups among young adults is low. This study aimed to investigate the factors affecting the intentions to receive health checkups among young adults in their 20s in Korea. The study design was a descriptive cross-sectional study and examined their intentions to receive health checkups, their health beliefs (perceived sensitivity, perceived severity, perceived benefit, perceived barrier, cue to action, self-efficacy), their attitude toward health checkups, and their knowledge of health checkups. The participants were 252 adults in their 20s in South Korea who were eligible for national health checkups. The intentions to receive check-ups model identified five variables, including sex, perceived sensitivity, cue to action, self-efficacy, and attitudes toward health checkups, were as significant influencing factors for the intentions to receive health checkups with 51.0% explanatory power (F = 53.18, *p* < 0.001). Different approaches must be adopted according to past experiences with health checkups when attempting to improve the intentions to receive health checkups in young adults.

## 1. Introduction

As medical technology advances and emphasis on preventive care emerges, it is critical to determine the probabilities of diseases early. In other words, early detection of previously undetected diseases or modifiable risk factors can reduce disease morbidity and mortality [1]. Regular health checkups allow early treatment when problems occur and prevent disease progression. Furthermore, health checkups can minimize individual economic loss and the national health insurance expenditure, ultimately lowering social costs [2].

Excluding healthcare for medical personnel and veterans, all residents of South Korea are covered by national health insurance [3]. Since 1980, South Korea has provided free health checkups to those with insurance. Later, the coverage was expanded to include dependents of the insured and now includes select cancer examinations in addition to regular health checkups [2]. However, this system mainly covers people over 40 years, while young adults in their 20s and 30s, excluding insured employees and self-employed householders, have been excluded for a long time.

Recently, the need of health checkup for young adults has been emerging as the prevalence of chronic diseases has been increasing in young adults [4] as well as the prevalence of depression, a preceding disease of suicide which is reported as a major cause of death in adults in their 20s and 30s [5]. Hence, general health checkups have become accessible to young adults aged 20 years or older since 2019 and are offered for free every two years [6]. However, since this is relatively new, the health checkup rate among adults in their 20s was 57% in males and 60.4% in females in 2020, indicating a high percentage of people who did not receive health checkups [4]. Furthermore, approximately 26.6% of adults in their 20s who received health checkups either had a suspected illness or were diagnosed with a disease during that period [4], indicating the need to examine young adults for proper health management.

Health Belief Model: The HBM was developed to explain why most people would not participate in programs for disease prevention and health checkups [7]. The HBM is an important predictive factor that can explain preventive health behaviors for certain diseases and various health-related behaviors, such as smoking, exercise, patient role, and use of medical services [8]. Health beliefs, defined as personal beliefs related to behaviors of perceiving and managing certain diseases, include perceived sensitivity, perceived severity, perceived benefit, perceived barrier, and cue to action [8].

According to a study examining the correlation between health promotion behaviors and health beliefs, health promotion behaviors had a positive correlation with perceived severity (*r* = 0.60) and perceived benefit (*r* = 0.55) and a negative correlation with perceived barriers (*r* = −0.32) [9]. As health checkups are one of the health promotion behaviors, individual health beliefs are likely to be a crucial factor influencing health promotion behaviors in young adults. Among global studies on the relationship between health beliefs and health checkups, a study elucidating factors that affect the intentions to receive health checkups in the elderly aged 60 or older in China reported that intentions to receive checkups decreased when people had lower self-efficacy, decreased awareness of the benefits of health checkups, and more perceived barriers [10].

To engage in health promotion behaviors, an intention to be healthy is required, and appropriate behaviors according to the intention must be followed [11]. Although the intention to be healthy and its execution in action are correlated, these differ. In other words, the intention to receive a health checkup differs from executing the intention. Therefore, the intentions to receive health checkups between people who actually received it and those who did not needs to be verified and improved. Although the execution rate of general health checkups is lower in adults in their 20s than in other age groups in South Korea, there are not enough studies elucidating the factors that affect the intentions and execution of receiving health examinations following the recent implementation of the current health checkup system. Hence, our study aimed to investigate the rates of health checkups in adults in their 20s and analyze the health beliefs, behaviors, and knowledge of the health checkup and non-health checkup groups to determine the factors affecting the intentions to undergo health checkups.

## 2. Materials and Methods

### 2.1. Study Design

We conducted a descriptive cross-sectional study to elucidate the factors that affect the intentions to receive health checkups depending on its execution. We determined whether adults in their 20s in South Korea had received health check-ups and divided them into health checkup and non-health checkup groups. We then examined their intentions to receive health checkups, their health beliefs (perceived sensitivity, perceived severity, perceived benefit, perceived barrier, cue to action, self-efficacy), their attitudes toward health checkups, and their knowledge of health checkups.

### 2.2. Participants

The participants in this study were adults in their 20s in South Korea who were eligible for a national health checkup. The inclusion criteria, considering that the participants were eligible for more than one health checkup, included adults aged between 21 and 29 who were locally insured, uninsured employees, and qualified recipients for medical care. Exclusion criteria included adults younger than 21 years or adults aged 30 or older, insured employees, and those hospitalized or isolated and therefore could not be examined during the study period. The number of samples was calculated using G*Power version 3.1.9.4. Multiple linear regression analysis with Cohen’s [12] medium effect size f^2^ = 0.15, α = 0.05, 1 − β = 0.95, and 19 prediction variables showed that the number of participants should be 217. Considering the 10% dropout rate for data collection from the online survey, we collected data from 256 participants. After excluding four participants whose employers provided health checkups, an exclusion criterion, the final data from 252 participants were analyzed.

### 2.3. Research Ethics

This study was approved by the institutional review board (no. CBNU 2022-0160). Data were collected using panels from an online survey provider to ensure the safety of the researchers and participants against COVID-19. On the first web page of the online survey, guidelines, including the objectives, procedures, and data collection method of the study, were clearly outlined. Participation was voluntary, and participants had the right to terminate or reject the survey. The contact information of the researchers was provided for any clarification, and it was explained that when participants decided to withdraw from participation, their responses would not be included in the data analysis. It was also explained that there would be no disadvantages to terminating or rejecting the survey.

### 2.4. Tools

#### 2.4.1. Participant Characteristics

The characteristics of the participants included age, sex, education, religion, residence, economic status, health status, stress level, history of health checkups, satisfaction with health checkups, and sufficiency of health management with a national health checkup.

#### 2.4.2. Intentions to Receive Health Checkups

The intention to receive a health checkup is a behavior plan taken to prevent or detect a disease early when symptoms have not yet appeared for the purpose of disease prevention [13]. This definition was used to describe the intentions to receive health checkups in this study. The intentions to undergo health checkups were measured using Ko’s instrument, which was validated in the current study by revising and adjusting the composition and vocabulary [14]. Cronbach’s α was 0.875 in Ko’s study and 0.800 in our study [14].

#### 2.4.3. Health Belief

The survey for health belief consisted of 30 items under six categories based on the health belief model: four items each for perceived sensitivity, perceived severity, perceived benefit, perceived barrier, and cue to action, and 10 items for self-efficacy. Each item was measured using a 5-point Likert scale ranging from 1 (‘strongly disagree’) to 5 (‘strongly agree’). Perceived sensitivity, perceived severity, perceived benefit, and perceived barrier were measured using a tool validated in Ko’s study [15]; cue to action was measured using a tool validated in Kim’s study [16], and self-efficacy was measured using a tool validated in Ha’s study [17]. Higher scores indicated better or more positive health beliefs for each item.

Perceived sensitivity refers to the awareness of one’s exposure to a certain disease and the level of one’s perception of the probability of having that disease [7]. Cronbach’s α was 0.837 in Ko’s study [15] and 0.730 in our study.

Perceived severity is the level of one’s perception of the severity of an outcome of a certain disease from both physical and social perspectives [7]. Cronbach’s α was 0.827 in Ko’s study [15] and 0.875 in our study.

Perceived benefit refers to the personal perception of the positive effects of countermeasures in reducing the risk or sensitivity to disease [7]. In the current study, we describe the level of perception of the benefits and usefulness of health checkups. Cronbach’s α was 0.746 in Ko’s study [15] and 0.771 in our study.

Perceived barriers refer to personal beliefs about psychological and environmental factors that interfere with advised health-related behaviors [18]. Cronbach’s α was 0.715 in Ko’s study [15] and 0.747 in our study.

Cue to action, a variable predictive behavior, does not refer to the external action itself but the individual’s internal will to perform external action [19]. The cues to action in our study included internal recognition and external stimuli (interaction in personal relationships, effects of mass media, and advice from healthcare providers). Cue to action was measured using four items revised and adjusted from Kim’s instrument [16]. The items include healthcare providers’ explanations on the importance of health checkups, health checkups of family or close acquaintances, recommendations from family or close acquaintances, and information from the mail. Cronbach’s α was not presented in the previous study but was 0.555 in our study.

Self-efficacy is the belief that one is capable of properly performing the recommended health behaviors [20]. It consists of expectation for efficacy, referring to confidence in performing behaviors needed for health, and expectation for effects, referring to the expectation that the suggested behaviors would prevent risks and bring desirable effects on health [20]. In this study, self-efficacy describes a belief in one’s capability to successfully take preventive action to obtain positive effects from health checkups. Cronbach’s α was 0.92 in Ha’s study [17] and 0.885 in our study.

#### 2.4.4. Attitude toward Health Checkups

Attitude toward health checkups is an evaluative emotional state in one’s favor or disfavor for a certain object and a tendency to consistently perform certain behaviors [21]. To measure attitudes toward health checkups, seven items were validated after revising and adjusting Lim’s instrument to fit our purpose [22]. Each item used a 5-point scale ranging from 1 (‘strongly disagree’) to 5 (‘strongly agree’). Among these, two reversed items were subjected to reverse conversion. Higher scores indicated a stronger attitude toward health checkups. Cronbach’s α was 0.80 in Lim’s study [22] and 0.800 in our study.

#### 2.4.5. Knowledge of Health Checkups

Knowledge of health checkups implies knowledge of the methods of health checkup, interpretation of results, item checklist, and disease [22]. In this study, 14 items revised and validated from the instrument developed by Lim [22] referencing the criteria for student health checkups, e-book on health care, and National Health Insurance Corporation websites were used. The items provided choices, ‘yes’, ‘no,’ and ‘I don’t know’, and were scored 1 for correct answers and 0 for wrong answers or ‘I don’t know’. The total scores ranged from 0 to 14, with higher scores indicating a higher knowledge of health checkups. Cronbach’s α was not presented in the previous study but was 0.673 in our study.

### 2.5. Data Collection

Recruitment notices for participants and URLs for online surveys were provided to online survey panel providers 3–7 August 2022, and data were collected after filling out the survey. Personal information was removed from the collected data, and only items needed for the study were coded. Data from 252 participants were delivered to the researchers. The recruitment notice for the online survey described the study objectives, content, survey method, participant rights, and confidentiality. Their informed consent for voluntary participation was also obtained. Compensation was offered to participants who completed the survey according to the policies of the online survey providers.

### 2.6. Statistical Analysis

The collected data were analyzed using IBM SPSS, version 26.0 (IBM, Armonk, NY, USA). Participant characteristics were analyzed using real numbers, percentages, means, and standard deviations (SD) of the descriptive statistics. The intentions to receive health checkups, health beliefs (perceived sensitivity, perceived severity, perceived benefit, perceived barrier, cue to action, and self-efficacy), attitudes toward health checkups, and knowledge of health checkups were analyzed using means, standard deviations, and minimum and maximum values. Differences in participant characteristics between the health checkup and non-health checkup groups were analyzed using an independent t-test and χ^2^ test, and differences between the variables of the two groups were analyzed using an independent *t*-test. Differences in the intentions to receive health checkups according to participant characteristics were analyzed using an independent t-test and one-way analysis of variance (ANOVA) for each group, whereas differences between or within the groups and the interactions between the groups and characteristics were analyzed using two-way ANOVA. Correlations between the intentions to receive health checkups and health beliefs (perceived sensitivity, perceived severity, perceived benefit, perceived barrier, cue to action, and self-efficacy), attitudes toward health checkups, and knowledge of health checkups were analyzed using Pearson’s correlation coefficients. For the factors affecting the intentions to receive health checkups, only the characteristics and variables showing significance in the one-way ANOVA were introduced to the model, and stepwise multiple regression analysis was used for model simplicity. Whether the assumption of multiple regression analysis was satisfied was analyzed using a residual plot, standardized residual, tolerance, variance inflation factors (VIF), and Durbin–Watson values. Statistical significance was <0.05.

## 3. Results

### 3.1. Participant Characteristics and Their Differences between the Health Checkup Group and Non-Health Checkup Group

There were 127 females (50.4%), 146 participants who were currently in school (57.9%), 183 participants without religion (72.6%), 226 participants residing in the city (89.7%), and 38 participants with a high economic status (15.0%). The mean age of participants was 24.25 (SD 2.45) years. The mean health status was 2.63 (SD 0.85), and the mean stress level was 3.47 (SD 0.82). The mean satisfaction level for a health checkup in 112 participants who had received a health checkup within the past two years (health checkup group) was 7.04 (SD 1.87), and those who believed that national health checkups are sufficient for health management were 2.69 (SD 0.95). Differences in participant characteristics between the health checkup and non-health checkup groups were found in terms of sex (χ^2^ = 8.59, *p* = 0.003) and health status (χ^2^ = 4.30, *p* = 0.038). There were no other differences in characteristics between the two groups (Table 1).

### 3.2. Intentions to Receive Health Checkups, Health Beliefs, Attitudes toward Health Checkups, and Knowledge of Health Checkups in the Health Checkup and Non-Health Checkup Groups

The intention to receive a health checkup was 3.72 (SD 0.70) among all the participants. For health beliefs, perceived benefit was the highest at 4.11 (SD 0.57), and perceived barrier was the lowest at 2.74 (SD 0.76). Attitudes toward health checkups were 3.97 (SD 0.55), and knowledge of health checkup was 0.59 (SD 0.18) among all the participants. In the health checkup group, the intentions to receive health checkups (t = 2.73, *p* = 0.007, 95%CI: 0.07, 0.41), cues to action (t = 3.32, *p* = 0.001, 95%CI: 0.11, 0.42), self-efficacy (t = 3.55, *p* < 0.001, 95%CI: 0.12, 0.44), and attitudes toward health checkups (t = 3.25, *p* = 0.001, 95%CI: 0.09, 0.36) were higher than those of the non-health checkup group, and perceived barrier (t = −5.31, *p* < 0.001, 95%CI: −0.67, −0.31) was lower in the health checkup group (Table 2).

### 3.3. Differences in the Intentions to Receive Health Checkups According to Participant Characteristics between the Health Checkup and Non-Health Checkup Groups

In all participants and the non-health checkup group, differences in the intentions to receive health checkups according to participant characteristics were only observed in sex with females scoring higher than males (t = 3.50, *p* = 0.001, 95%CI: −0.48, −0.13; t = 2.30, *p* = 0.023, 95%CI: −0.50, −0.04). In the health checkup group, the intentions to receive health checkups according to participant characteristics was higher in females than in males (t = 2.13, *p* = 0.035, 95%CI: −0.52, −0.02) and was higher in participants with a higher economic status than those with a low economic status (F = 3.58, *p* = 0.031, 95%CI: 3.73, 3.98). The differences between the health checkup and non-health checkup groups according to participant characteristics were age (F = 6.61, *p* = 0.011, 95%CI: 3.60, 3.91), sex (F = 4.65, *p* = 0.032, 95%CI: 3.52, 3.96), education (F = 7.11, *p* = 0.008, 95%CI: 3.50, 3.99), religion (F = 5.04, *p* = 0.026, 95%CI: 3.49, 3.99), economic status (F = 11.18, *p* = 0.001, 95%CI: 3.48, 4.08), health status (F = 6.03, *p* = 0.015, 95%CI: 3.52, 3.99), and stress level (F = 7.32, *p* = 0.007, 95%CI: 3.50, 3.99). Differences within items between the two groups were found only in sex (F = 9.23, *p* = 0.003), and there was no interaction between the groups and characteristics (Table 3).

### 3.4. Correlations among the Intentions to Receive Checkups, Health Beliefs, Attitudes toward Health Checkups, and Knowledge of Health Checkup in the Health Checkup and Non-Health Checkup Groups

For all participants, the intentions to receive health checkups showed negative correlations only with perceived barrier among the health belief variables (r = −0.16, *p* = 0.010). It was positively correlated with the rest of the health belief variables, attitudes toward health checkups (r = 0.57, *p* < 0.001), and knowledge of health checkups (r = 0.19, *p* = 0.003). In the health checkup group, the intentions to receive health checkups was positively correlated with perceived sensitivity (r = 0.27, *p* = 0.004), perceived benefit (r = 0.45, *p* < 0.001), cue to action (r = 0.35, *p* < 0.001), self-efficacy (r = 0.54, *p* < 0.001), and attitudes toward health check-up (r = 0.51, *p* < 0.001) among the health belief variables. In the non-health checkup group, the intentions to receive health checkups showed positive correlations with the health belief variables, except for perceived barriers and attitudes toward health checkups (r = 0.60, *p* < 0.001) and knowledge of health check-ups (r = 0.19, *p* = 0.024) (Table 4).

### 3.5. Factors Affecting the Intentions to Receive Health Checkps in the Health Checkup Group and Non-Health Checkup Group

The participants, young adults in their 20s in South Korea, were divided into a health checkup group and a non-health checkup group depending on whether they had received a health checkup in the past 2 years, and multiple linear regression analysis was conducted to examine the factors affecting the intentions to receive health checkups in each group (Table 5). Only statistically significant variables from one-way ANOVA were selected and eliminated by 0.05 and 0.10 significance levels, respectively, using a stepwise method, and a model for the intentions to receive health checkups was established for each participant. The tolerance of the intentions to health checkups model was ≥0.1, and VIF was ≤10, showing no multicollinearity, and Durbin–Watson was close to 2.0, securing normality of residuals.

To establish a model for the intentions to receive health checkups for all participants, sex was assigned with dummy inputs, and continuous variables were entered for health belief variables, attitudes toward health checkups, and knowledge of health checkups using a stepwise method. The intentions to receive check-ups were lower in males than in females and were higher when perceived sensitivity, cue to action, self-efficacy, and attitudes toward health checkups were higher. Hence, five variables, including sex, perceived sensitivity, cue to action, self-efficacy, and attitudes toward health checkups, were determined as significant influencing factors for the intentions to receive health checkups with 51.0% explanatory power (F = 53.18, *p* < 0.001).

To establish a model for the intentions to receive health checkups in the health checkup group, sex was assigned with dummy inputs, and continuous variables were entered for economic status, perceived sensitivity, perceived benefit, cue to action, self-efficacy, and attitudes toward health checkups using a stepwise method. In the health checkup group, four variables, including sex, perceived sensitivity, self-efficacy, and attitudes toward health checkups, were determined as significant influencing factors for the intentions to receive health checkups with 43.0% explanatory power (F = 21.94, *p* < 0.001).

To establish the intentions to receive the health checkups model in the non-health checkup group, sex was assigned with dummy inputs, and continuous variables were entered for perceived sensitivity, perceived severity, perceived benefit, cue to action, self-efficacy, attitudes toward health checkups, and knowledge of health checkups using a stepwise method. In the non-health checkup group, four variables, including perceived sensitivity, perceived benefit, self-efficacy, and attitudes toward health checkups, were determined as significant influencing factors for the intentions to receive health checkups with 53.4% explanatory power (F = 40.77, *p* < 0.001).

## 4. Discussion

This study was conducted to elucidate the factors affecting the intentions to receive health checkups in the health checkup and non-health checkup groups among South Korean adults in their 20s. Among health beliefs, attitudes toward health checkups and knowledge of health checkups were statistically significant variables obtained from one-way ANOVA univariate analysis used to determine the factors affecting the intentions to receive health checkups in the health checkup group, non-health checkup group, and all participants. The results showed a high explanatory power, ranging from 43.0% to 53.4%. Hence, the variables examined in this study were verified as significant to explain the intentions to receive health checkups in young adults. The factors affecting the intentions to receive health checkups in all participants were sex, perceived sensitivity, cue to action, self-efficacy, and attitudes toward health checkups. However, the influencing factors were different between the health checkup and non-health checkup groups, and they also showed different patterns compared to the entire group.

The factors affecting the intentions to receive health checkups were sex, perceived sensitivity, self-efficacy, and attitudes toward health checkups in the health checkup group and perceived severity, perceived benefit, self-efficacy, and attitudes toward health checkups in the non-health checkup group. In other words, the factors determining intentions varied depending on past health checkup experience. This suggests that strategies to promote health checkups should consider the past experiences of participants. According to a previous study on predictive factors for the intentions to receive vaccination and for preventive behaviors, such as receiving a health checkup, it was observed that the presence or absence of recent related behaviors was a major predictive factor for the intentions, which are in line with our study results [23]. Therefore, when planning interventions to improve the execution rate of health checkups, healthcare providers should examine the past checkup behaviors of the subjects and utilize different approaches depending on the presence or absence of related behaviors.

Sex was selected as an important factor determining intentions not only in all participants but also in the health checkup group. This is similar to the findings from a previous study examining the factors affecting health intentions in participants of health checkups in Japan, which verified that age and sex were crucial influencing factors [24]. Therefore, considering the background of participants, such as age and sex, is necessary when promoting health checkups in young adults. As the intentions to receive health checkups were lower in males than in females, strategies to target males are required.

Health beliefs, such as perceived sensitivity, perceived severity, and perceived benefit, were major factors determining the intentions to receive health checkups among adults in their 20s. Perceived sensitivity affected intentions in the health checkup group, which is in line with the results of a previous study examining factors influencing the intentions to undergo health examinations in adults in Taiwan [25]. However, unlike the health checkup group, perceived severity and perceived benefit were important factors in the non-health checkup group, demonstrating different trends between these two groups. According to a previous study on the health behavior decision-making process implemented in the users of online health communities who did not receive the human papillomavirus (HPV) vaccine, perceived sensitivity and perceived severity both affected the health behavioral intentions, which is different from our study result [26]. Xu [26] reported that perceived sensitivity and perceived severity affected health behavioral intentions directly and indirectly through communication behaviors. Although health beliefs were determined as factors affecting behaviors and intentions in previous studies, the results should be interpreted carefully, as sub-variables showed variances according to the studies. However, changes in health beliefs must precede changes in an individual’s health intentions.

Self-efficacy and attitudes toward health checkups were the influencing factors that were essential not only in all participants but also in the health checkup and non-health checkup groups. This finding is consistent with the results of previous studies. According to a meta-analysis examining the effects of attitudes and self-efficacy on health-related intentions and behaviors, self-efficacy and attitudes both resulted in medium changes in intentions [27]. In other words, interventions that alter self-efficacy or attitudes toward health can lead to changes in behaviors and intentions for individual health behaviors. Therefore, individual attitudes and self-efficacy should be verified when designing interventions to improve the execution rate of health checkups among young adults.

According to the structural model of behavioral health intentions proposed by Huang et al. [25], self-efficacy has direct effects on intentions but can also have indirect effects by substantially affecting perceived benefits and perceived barriers. Hence, improving self-efficacy would be a good strategy to increase health checkups in young adults. Although it was conducted on children, a study reported that those with an intention–behavior gap with health behaviors having lower self-efficacy than those with high intentions, high behaviors, and high self-efficacy than those with low intentions and low behaviors [28]. In other words, self-efficacy is likely to reduce the intention–behavior gap. Therefore, interventions that emphasize self-efficacy are needed to improve health checkup intentions in the non-health checkup group.

These results suggest that the intentions to undergo health checkups in young adults are affected by various psychosocial factors. In addition, the influencing factors varied according to past health checkup experiences. Therefore, to increase the rate of health checkups in young adults, past experiences with health checkups should be examined, and different strategies need to be adapted accordingly. Furthermore, since self-efficacy and attitudes toward health checkups both affect the intentions to receive health checkups regardless of past experiences, interventions that increase self-efficacy and promote positive attitudes are needed. This study has several limitations. First, key factors potentially influencing an individual’s decision to undergo a health checkup should be investigated, including whether the subject has a family history of a disease, such as cancer or the presence of depression and a late-onset genetic disorder. Second, this study was a cross-sectional study to identify factors affecting intentions of the health checkups. Since it was not a cohort study in which two groups were observed over a long period of time, it was careful to draw causal conclusions about whether the influencing factors in this study directly affect the intentions of the health examinations. Therefore, the results of this study should be carefully interpreted and applied to practice and policy.

## 5. Conclusions

This study was conducted to examine the factors affecting the intentions to receive health checkups in young adults in South Korea, who were divided into health checkup and non-health checkup groups. The factors affecting the intentions to receive health checkups were sex, perceived sensitivity, self-efficacy, and attitudes toward health checkups in the health checkup group and perceived severity, perceived benefit, self-efficacy, and attitudes toward health checkups in the non-health checkup group. As the factors affecting the intentions to receive health checkups differed between the two groups, different approaches must be adopted according to past experiences with health checkups when attempting to improve the intentions to receive health checkups in young adults. Furthermore, strategies to improve self-efficacy and promote positive attitudes are required regardless of past health checkup experiences.

## Figures and Tables

**Table 1:** Characteristics of the participants (n = 252).

Characteristics		Total (n = 252)	Health Checkup Group (n = 112)	Non-Health Checkup Group (n = 140)	t or χ^2^ (*p*)
		n (%)			
Age (year)	<25	169 (67.1)	79 (70.5)	90 (64.3)	1.10 (0.294)
	≥26	83 (32.9)	33 (29.5)	50 (35.7)	
	M ± SD	24.25 ± 2.45	24.13 ± 2.43	24.34 ± 2.47	−0.65 (0.517)
Sex	Female	127 (50.4)	68 (60.7)	59 (42.1)	8.59 (0.003)
	Male	125 (49.6)	44 (39.3)	81 (57.9)	
Education	in college	146 (57.9)	70 (62.5)	76 (54.3)	1.72 (0.189)
	Graduate	106 (42.1)	42 (37.5)	64 (45.7)	
Religion	No	183 (72.6)	77 (68.8)	106 (75.7)	1.52 (0.218)
	Yes	69 (27.4)	35 (31.3)	34 (24.3)	
Residence	City	226 (89.7)	98 (87.5)	128 (91.4)	1.04 (0.308)
	Rural	26 (10.3)	14 (12.5)	12 (8.6)	
Economic status	High	38 (15.0)	17 (15.2)	21 (15.0)	0.01 (0.999)
	Middle	108 (42.9)	48 (42.9)	60 (42.9)	
	Low	106 (42.1)	47 (42.0)	59 (42.1)	
	M ± SD	3.35 ± 0.82	3.32 ± 0.92	3.37 ± 0.91	−0.43 (0.667)
Health status	<2.63	110 (43.7)	57 (50.9)	53 (37.9)	4.30 (0.038)
	≥2.63	142 (56.3)	55 (49.1)	87 (62.1)	
	M ± SD	2.63 ± 0.85	2.55 ± 0.85	2.69 ± 0.85	−1.30 (0.196)
Stress level	<3.47	122 (48.4)	58 (51.8)	64 (45.7)	0.92 (0.338)
	≥3.47	130 (51.6)	54 (48.2)	76 (54.3)	
	M ± SD	3.47 ± 0.82	3.45 ± 0.75	3.49 ± 0.88	−0.45 (0.650)
Satisfaction of health checkup	<7.04		62 (55.4)		
	≥7.04		50 (44.6)		
	M ± SD		7.04 ± 1.87		
sufficiency of health management with a national health checkup	<2.69		57 (50.9)		
	≥2.69		55 (49.1)		
	M ± SD		2.69 ± 0.95		

Notes. N: number, M: mean, SD: standard deviation, t: independent *t*-test, χ^2^: chi-square test.

**Table 2:** Descriptive statistics of the variables (n = 252).

Variables	Total (n = 252)		Health Checkup Group (n = 112)		Non-Health Checkup Group (n = 140)		t (*p*)
	M ± SD	Min.~Max.	M ± SD	Min.~Max.	M ± SD	Min.~Max.	
Intention to health checkup	3.72 ± 0.70	1.75~5.00	3.86 ± 0.60	1.50~5.00	3.62 ± 0.72	1.75~5.00	2.73 (0.007)
Perceived sensitivity	3.47 ± 0.70	1.00~5.00	3.51 ± 0.69	1.00~5.00	3.44 ± 0.71	1.00~5.00	0.81 (0.420)
Perceived severity	3.28 ± 0.83	1.00~5.00	3.27 ± 0.87	2.50~5.00	3.29 ± 0.81	1.00~5.00	−0.19 (0.853)
Perceived benefit	4.11 ± 0.57	2.50~5.00	4.17 ± 0.55	1.00~4.25	4.07 ± 0.58	2.50~5.00	1.33 (0.186)
Perceived barrier	2.74 ± 0.76	1.00~5.00	2.47 ± 0.75	2.50~5.00	2.95 ± 0.70	1.00~5.00	−5.31 (<0.001)
Cue to action	3.60 ± 0.64	1.75~5.00	3.75 ± 0.60	1.50~5.00	3.48 ± 0.65	1.75~5.00	3.32 (0.001)
Self-efficacy	3.56 ± 0.64	1.50~5.00	3.72 ± 0.60	2.00~5.00	3.44 ± 0.64	1.70~5.00	3.55 (<0.001)
Attitude toward health checkup	3.97 ± 0.55	2.29~5.00	4.10 ± 0.56	2.71~5.00	3.87 ± 0.53	2.29~5.00	3.25 (0.001)
Knowledge of health checkup	0.59 ± 0.18	0.07~1.00	0.60 ± 0.18	0.07~0.93	0.58 ± 0.19	0.07~1.00	0.87 (0.384)

Notes. M: mean, SD: standard deviation, Min.: minimum, Max.: maximum.

**Table 3:** Differences in intentions to health checkups according to participants’ characteristics.

Characteristics		Total (n = 252)		Health Checkup Group (n = 112)		Non-Health Checkup Group (n = 140)			F (*p*)
		M ± SD	t or F (*p*)	M ± SD	t or F (*p*) (Scheffe)	M ± SD	t or F (*p*)		
Age (year)	<25	3.72 ± 0.69	-0.09 (0.928)	3.85 ± 0.67	−0.22 (0.825)	3.61 ± 0.70	-0.17 (0.865)	Group	6.61 (0.011)
	≥26	3.73 ± 0.73		3.88 ± 0.65		3.63 ± 0.77		Age	0.08 (0.783)
								Group × Age	0.01 (0.962)
Sex	Female	3.87 ± 0.64	3.50 (0.001)	3.96 ± 0.67	2.13 (0.035)	3.77 ± 0.60	2.30 (0.023)	Group	4.65 (0.032)
	Male	3.57 ± 0.73		3.69 ± 0.63		3.50 ± 0.78		Sex	9.23 (0.003)
								Group × Sex	0.00 (0.991)
Education	in college	3.76 ± 0.65	1.00 (0.321)	3.87 ± 0.62	0.29 (0.770)	3.66 ± 0.66	0.81 (0.421)	Group	7.11 (0.008)
	Graduate	3.67 ± 0.78		3.83 ± 0.74		3.56 ± 0.79		Education	0.58 (0.449)
								Group × Education	0.11 (0.737)
Religion	No	3.71 ± 0.69	−0.32 (0.749)	3.87 ± 0.63	0.23 (0.819)	3.60 ± 0.71	−0.36 (0.723)	Group	5.04 (0.026)
	Yes	3.75 ± 0.75		3.84 ± 0.75		3.65 ± 0.75		Religion	0.01 (0.922)
								Group × Religion	0.17 (0.680)
Residence	City	3.74 ± 0.70	0.82 (0.411)	3.89 ± 0.63	1.29 (0.198)	3.62 ± 0.72	0.16 (0.870)	Group	1.29 (0.258)
	Rural	3.62 ± 0.79		3.64 ± 0.86		3.58 ± 0.73		Residence	0.94 (0.333)
								Group × Residence	0.52 (0.471)
Economic status	High ^a^	3.88 ± 0.74	1.33 (0.265)	4.24 ± 0.63	3.58 (0.031) (a > b, c)	3.58 ± 0.70	0.16 (0.851)	Group	11.18 (0.001)
	Middle ^b^	3.66 ± 0.70		3.75 ± 0.69		3.59 ± 0.70		Economic status	1.69 (0.187)
	Low ^c^	3.73 ± 0.70		3.83 ± 0.61		3.66 ± 0.76		Group ×Economic status	1.95 (0.144)
Health status	<2.63	3.80 ± 0.76	1.44 (0.152)	3.86 ± 0.70	0.04 (0.968)	3.73 ± 0.82	1.34 (0.184)	Group	6.03 (0.015)
	≥2.63	3.67 ± 0.65		3.85 ± 0.63		3.55 ± 0.64		Health status	1.04 (0.308)
								Group × Health status	0.93 (0.336)
Stress level	<3.47	3.75 ± 0.69	0.58 (0.560)	3.91 ± 0.68	0.79 (0.430)	3.61 ± 0.68	−0.10 (0.920)	Group	7.32 (0.007)
	≥3.47	3.70 ± 0.72		3.81 ± 0.65		3.62 ± 0.76		Stress level	0.24 (0.623)
								Group × stress level	0.40 (0.528)
Satisfaction of health checkup	<7.04			4.08 ± 0.66	3.33 (0.001)				
	≥7.04			3.68 ± 0.61					
sufficiency of health management with a national health checkup	<2.69			3.91 ± 0.69	0.97 (0.337)				
	≥2.69			3.80 ± 0.63					

Notes. M: mean, SD: standard deviation, post hoc: Scheffe test. ^a^: high level of economic status, ^b^: middle level of economic status, ^c^: low level of economic status.

**Table 4:** Correlation among the variables (n = 252).

Group	Variables	Intention to Health Checkup	Perceived Sensitivity	Perceived Severity	Perceived Benefit	Perceived Barrier	Cue to Action	Self-Efficacy	Attitude Toward Health Checkup
		r (*p)*							
Total (n = 252)	Perceived sensitivity	0.35 (<0.001)	1						
	Perceived severity	0.25 (<0.001)	0.67 (<0.001)	1					
	Perceived benefit	0.55 (<0.001)	0.29 (<0.001)	0.17 (0.009)	1				
	Perceived barrier	−0.16 (0.010)	0.23 (<0.001)	0.20 (0.002)	−0.25 (<0.001)	1			
	Cue to action	0.44 (<0.001)	0.29 (<0.001)	0.28 (<0.001)	0.47 (<0.001)	−0.15 (0.015)	1		
	Self-efficacy	0.57 (<0.001)	0.12 (0.058)	0.04 (0.536)	0.51 (<0.001)	−0.32 (<0.001)	0.41 (<0.001)	1	
	Attitude toward health checkup	0.57 (<0.001)	0.21 (0.001)	0.13 (0.033)	0.68 (<0.001)	−0.39 (<0.001)	0.32 (<0.001)	0.47 (<0.001)	1
	Knowledge of health checkup	0.19 (0.003)	0.14 (0.031)	0.09 (0.166)	0.20 (0.002)	−0.08 (0.221)	0.30 (<0.001)	0.20 (0.001)	0.24 (<0.001)
Health checkup group (n = 112)	Perceived sensitivity	0.27 (0.004)	1						
	Perceived severity	0.17 (0.076)	0.61 (<0.001)	1					
	Perceived benefit	0.45 (<0.001)	0.16 (0.089)	0.11 (0.232)	1				
	Perceived barrier	−0.18 (0.060)	0.15 (0.119)	0.03 (0.738)	−0.32 (0.001)	1			
	Cue to action	0.35 (<0.001)	0.18 (0.059)	0.16 (0.092)	0.48 (<0.001)	−0.19 (0.047)	1		
	Self-efficacy	0.54 (<0.001)	0.10 (0.276)	0.07 (0.470)	0.49 (<0.001)	−0.36 (<0.001)	0.37 (<0.001)	1	
	Attitude toward health checkup	0.51 (<0.001)	0.03 (0.724)	0.06 (0.530)	0.68 (<0.001)	−0.39 (<0.001)	0.26 (0.007)	0.47 (<0.001)	1
	Knowledge of health checkup	0.16 (0.085)	−0.02 (0.848)	−0.02 (0.805)	0.21 (0.026)	−0.18 (0.054)	0.14 (0.141)	0.23 (0.017)	0.30 (0.001)
Non-health checkup group (n = 140)	Perceived sensitivity	0.41 (<0.001)	1						
	Perceived severity	0.33 (<0.001)	0.73 (<0.001)	1					
	Perceived benefit	0.61 (<0.001)	0.38 (<0.001)	0.21 (0.013)	1				
	Perceived barrier	−0.07 (0.439)	0.35 (<0.001)	0.36 (<0.001)	−0.16 (0.059)	1			
	Cue to action	0.47 (<0.001)	0.36 (<0.001)	0.39 (<0.001)	0.45 (<0.001)	−0.02 (0.809)	1		
	Self-efficacy	0.56 (<0.001)	0.12 (0.169)	0.02 (0.799)	0.52 (<0.001)	−0.20 (0.017)	0.39 (<0.001)	1	
	Attitude toward health checkup	0.60 (<0.001)	0.35 (<0.001)	0.21 (0.012)	0.68 (<0.001)	−0.31 (<0.001)	0.32 (<0.001)	0.43 (<0.001)	1
	Knowledge of health checkup	0.19 (0.024)	0.24 (0.004)	0.18 (0.036)	0.18 (0.033)	0.03 (0.719)	0.40 (<0.001)	0.18 (0.038)	0.18 (0.029)

Notes. r: Pearson correlation coefficient.

**Table 5:** Influencing factors on intentions to health checkups (n = 252).

Variables	Total (n = 252)		Health Checkup Group (n = 112)		Non-Health Checkup Group (n = 140)	
	*β*	t (*p*)	*β*	t (*p*)	*β*	t (*p*)
Intercept		−1.08 (0.280)		0.46 (0.647)		−2.22 (0.028)
Sex (male)	−0.10	−2.17 (0.031)	−0.17	−2.38 (0.019)		
Perceived sensitivity	0.19	4.13 (<0.001)	0.20	2.68 (0.009)		
Perceived severity					0.22	3.65 (<0.001)
Perceived benefit					0.22	2.56 (0.012)
Cue to action	0.12	2.36 (0.019)				
Self-efficacy	0.35	6.49 (<0.001)	0.38	4.62 (<0.001)	0.33	4.85 (<0.001)
Attitudes toward health checkups	0.31	6.01 (<0.001)	0.31	3.84 (<0.001)	0.26	3.27 (0.001)
F (*p*)	53.18 (<0.001)		21.94 (<0.001)		40.77 (<0.001)	
Adj. R^2^ (%)	51.0		43.0		53.4	
Tolerance	0.68~0.91		0.76~0.97		0.47~0.93	
VIF	1.10~1.46		1.03~1.32		1.07~2.11	
Durbin–Watson	1.87		2.29		1.73	

Notes. *β*: standardized regression coefficient, VIF: variance inflation factor.

## Data Availability

Not applicable.
